# The incidence of post-intubation hypertension and association with repeated intubation attempts in the emergency department

**DOI:** 10.1371/journal.pone.0212170

**Published:** 2019-02-11

**Authors:** Akihiko Inoue, Hiroshi Okamoto, Toru Hifumi, Tadahiro Goto, Yusuke Hagiwara, Hiroko Watase, Kohei Hasegawa, for the Japanese Emergency Medicine Network Investigators

**Affiliations:** 1 Department of Emergency and Critical Care Medicine, Hyogo Emergency Medical Center, Kobe, Hyogo, Japan; 2 Faculty of Medicine, Graduate School of Medicine, Kagawa University, Miki, Kita, Kagawa, Japan; 3 Center for Clinical Epidemiology, St. Luke’s International University, Chuo-ku, Tokyo, Japan; 4 Emergency and Critical Care medicine, St. Luke's International Hospital, Chuo-ku, Tokyo, Japan; 5 Department of Emergency Medicine, Massachusetts General Hospital, Boston, Massachusetts, United States of America; 6 Department of Pediatric Emergency and Critical Care Medicine, Tokyo Metropolitan Children's Medical Center, Fuchu, Tokyo, Japan; 7 Department of Surgery, University of Washington, Seattle, Washington, United States of America; 8 Harvard Medical School, Boston, Massachusetts, United States of America; San Gerardo Hospital, ITALY

## Abstract

**Background:**

Studies in the non-emergency department (ED) settings have reported the relationships of post-intubation hypertension with poor patient outcomes. While ED-based studies have examined post-intubation hypotension and its sequelae, little is known about, post-intubation hypertension and its risk factors in the ED settings. In this context, we aimed to identify the incidence of post-intubation hypertension in the ED, and to test the hypothesis that repeated intubation attempts are associated with an increased risk of post-intubation hypertension.

**Methods:**

This study is a secondary analysis of the data from a multicenter prospective observational study of emergency intubations in 15 EDs from 2012 through 2016. The analytic cohort comprised all adult non-cardiac-arrest patients undergoing orotracheal intubation without pre-intubation hypotension. The primary exposure was the repeated intubation attempts, defined as ≥2 laryngoscopic attempts. The outcome was post-intubation hypertension defined as an increase in systolic blood pressure (sBP) of >20% along with a post-intubation sBP of >160 mmHg. To investigate the association of repeated intubation attempts with the risk of post-intubation hypertension, we fit multivariable logistic regression models adjusting for ten potential confounders and patient clustering within the EDs.

**Results:**

Of 3,097 patients, the median age was 69 years, 1,977 (64.0%) were men, and 991 (32.0%) underwent repeated intubation attempts. Post-intubation hypertension was observed in 276 (8.9%). In the unadjusted model, the incidence of post-intubation hypertension did not differ between the patients with single intubation attempt and those with repeated attempts (8.5% versus 9.8%, unadjusted P = 0.24). By contrast, after adjusting for potential confounders and patient clustering in the random-effects model, the patients who underwent repeated intubation attempts had a significantly higher risk of post-intubation hypertension (OR, 1.56; 95% CI, 1.11–2.18; adjusted P = 0.01).

**Conclusions:**

We found that 8.9% of patients developed post-intubation hypertension, and that repeated intubation attempts were significantly associated with a significantly higher risk of post-intubation hypertension in the ED.

## Introduction

Tracheal intubation–the definitive management for securing the airway–is one of the most invasive procedures performed in the emergency department (ED). Tracheal intubation in the ED is a high-risk procedure due to the urgency of the situation, limited time for preparation, and unstable patient’s condition. Indeed, the literature has reported the incidence of intubation–related adverse events of 12%-26% in the ED [1–3]. Emerging evidence have also shown that repeated intubation attempts are associated with an increased risk of adverse events in the ED [4–8].

Among the various intubation-related adverse events in the ED (e.g., cardiac arrest, hypoxemia, esophageal intubation with delayed recognition, and hemodynamic compromise [1, 6]), most studies have investigated post-intubation hypotension and its sequelae [1, 9–11]. Within the sparse literature, studies in *non*-ED settings (e.g., operation room, intensive care unit) have reported that post-intubation hypertension is associated with myocardial ischemia [12, 13], re-rupture of aneurysmal subarachnoid hemorrhage [14], and poor outcomes in patients with severe traumatic brain injury [15], suggesting that post-intubation hypertension should also be considered as an important adverse event. While ED airway management has unique characteristics–e.g., limited patient’s physiologic reserve and potentially limited resources, no study has examined its incidence or the relationship with multiple intubation attempts as a risk factor in the ED setting.

To address the knowledge gap in the literature, by using the data from a prospective multicenter study of ED airway management, we aimed to examine the incidence of post-intubation hypertension, and test the hypothesis that repeated intubation attempts are associated with a higher risk of post-intubation hypertension in the ED.

## Materials and methods

### Study design and setting

This study was a secondary analysis of the data from the second Japanese Emergency Airway Network (JEAN-2) Study–a multicenter prospective observational study designed to characterize the current ED airway management and outcomes across Japan. The study design, setting, methods of measurement, and measured variables have been reported previously [3, 6, 16–19]. In brief, JEAN-2 is a consortium of 15 academic and community medical centers from different geographic regions across Japan. All 15 EDs were staffed by emergency attending physicians and had an affiliation with emergency medicine residency training program. Participating institutions included level I (n = 12) or level II (n = 3) equivalent trauma centers with a median ED census of 27,000 visits per year (range, 1,000–65,000 visits). Each ED maintained individual protocols, policies, and procedures for ED airway management. Intubations were performed by attending physicians or by resident physicians at the discretion of supervising ED attending physicians. The institutional review board of Fukui University Hospital, Fukui Prefectural Hospital, Kameda Medical Center, Kurashiki Central Hospital, Nagoya Ekisaikai Hospital, Nigata City General Hospital, Okinawa Chubu Prefectural Hospital, Otowa Hospital, Shonan Kamakura General Hospital, St Marianna University School of Medicine Hospital, Tokyo Bay Urayasu Ichikawa Medical Center, University Hospital, Kyoto Prefectural University of Medicine, Yokohama Rosai Hospital, Kishiwada Tokushukai Hospitals, Hyogo Emergency Medical Center, and Massachusetts General Hospital approved the protocol with waiver of informed consent prior to the data collection.

### Study participants

In the current analysis, we included consecutive adult patients who were intubated in the ED from February 2012 through November 2016. We excluded patients with cardiac arrest, those with systolic blood pressure (sBP) of <90 mmHg before first intubation attempt, those with missing data on age or blood pressure, pediatric patients (aged <18 years), and those who underwent nasotracheal intubation or surgical cricothyrotomy.

### Data collection and variables

Immediately after each intubation, intubator used a standardized data collection form to record the data, including age, sex, estimated body mass index (BMI), primary indication for intubation, methods of intubation, modified LEMON score [18], medications and dosage (e.g., sedatives, neuromuscular blockades), devices, specialty of the intubators, number of intubation attempts, adverse events, and pre- and post-intubation vital signs. Methods of intubation were categorized to rapid sequence intubation (RSI) and non-RSI. RSI was defined as intubation with virtually simultaneous administration of a sedative and rapidly acting neuromuscular blocking agent [20]. Specialty of the intubators categorized as transitional year resident (post-graduate year 1 or 2), emergency medicine resident, emergency medicine attending physician, and others. Blood pressure was measured at immediately before first intubation attempt (pre-intubation BP), immediately after successful intubation (post-intubation BP) and 30 minutes after intubation, by using noninvasive blood pressure or invasive arterial pressure monitoring.

### Primary exposure

The exposure of interest was repeated intubation attempts, defined as ≥2 laryngoscopic attempts for a single patient encounter [7, 8, 21]. An intubation “attempt” was defined as a single insertion of the laryngoscope (or other devices) past the teeth [6].

### Outcome measure

The outcome measure of interest was post-intubation hypertension, defined as a >20% increase in sBP compared to pre-intubation sBP along with a post-intubation sBP of >160 mmHg, according to prior studies [4, 22–26]. The literature has documented that these post-intubation hypertension events are associated with poor outcome, such as myocardial ischemia [13, 27]. re-rupture of aneurysmal subarachnoid hemorrhage [28], unfavorable neurological outcome [15], propagation of aortic dissection, rupture of aneurysmal thoracic or abdominal, and accelerated bleeding in trauma patients [29]. The percent change in sBP–an index of hemodynamic instability (or stability)–was calculated as (post-intubation sBP–pre-intubation sBP) / pre-intubation sBP × 100 [30].

### Statistical analysis

We first compared the patient characteristics and airway management characteristics according to the primary outcome (post-intubation hypertension vs. no post-intubation hypertension groups) by using Mann–Whitney *U* test for continuous variables and chi-squared test for categorical variables. Next, to test the study hypothesis that repeated intubation attempts (the primary exposure) are associated with the risk of post-intubation hypertension (the outcome), we constructed unadjusted and adjusted logistic regression models. In the multivariable model, we adjusted for potential confounders, including age, sex, BMI, indication of intubation, modified LEMON score, device of intubation, premedication, sedatives, neuromuscular blockades, and specialty of the intubator [4, 29]. As recommended by clinical epidemiologists and statisticians [31, 32], we have selected the covariates based on the clinical plausibility and *a priori* knowledge. Additionally, to account for patient clustering within the EDs, we also constructed random-effects models with binomial response using random intercepts for the EDs. To examine the robustness of our inference, in the sensitivity analyses, we used a different cut-off for the number of intubation attempts (≤2 vs. ≥3 vs. intubation attempts), modeled the number of attempts as an ordinal variable (instead of a binary variable), modeled the number of attempts as a categorical variable, and repeated the analysis in the patients who underwent RSI and in those who were intubated with sedatives (i.e., excluded patients without sedative use). All analyses were performed using STATA 14 (StataCorp, College Station, TX) and JMP statistical software (version 12; SAS Institute, Cary, NC, USA). A two-sided P value of <0.05 was considered statistically significant for all analyses.

## Results

**Fig 1:**
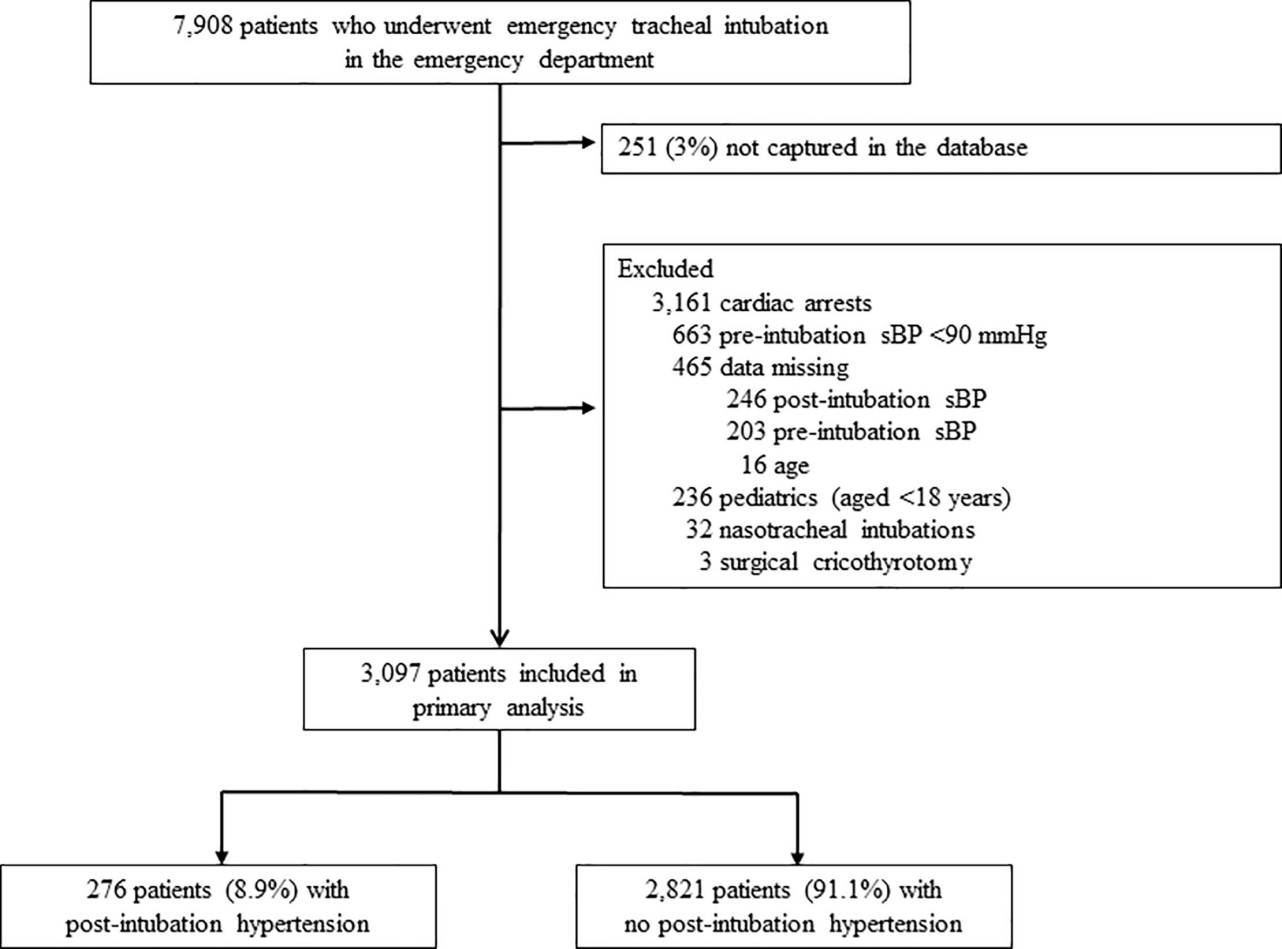
Patients receiving tracheal intubation in the emergency department. Abbreviation: sBP, systolic blood pressure.

During the 58-month period, there were 7,908 patients who underwent emergency airway management in the EDs. Among these, 7,657 patients were recorded in the database (capture rate, 97%). We excluded 3,161 patients with cardiac arrest, 663 patients with pre-intubation sBP of <90 mmHg, 465 patients with missing data on age or BP (246 data missing on post-intubation sBP, 203 data missing on pre-intubation sBP, and 16 data missing on age), 236 pediatric patients, and 35 patients who underwent nasotracheal intubation or surgical cricothyrotomy. The remaining 3,097 patients comprised the analytic cohort (Fig 1).

Blood pressure was measured at immediately before first intubation attempt (pre-intubation sBP), immediately after successful intubation (post-intubation sBP) and 30 minutes after intubation, by using noninvasive blood pressure or invasive arterial pressure monitoring.

Overall, the median age was 69 years (IQR 53–78 years), 64.0% were men, and 84.2% of intubations involved medical emergencies (Table 1). Of these, post-intubation hypertension was observed in 8.9% of patients (n = 276). A comparison of the patient characteristics between the patients who developed post-intubation hypertension and those who did not were shown in Tables 1 and 2. Overall, 68.0% were successfully intubated at the first intubation attempt while 32.0% underwent repeated intubation attempts (median 2 attempts; IQR 2–3 attempts). The patient characteristics and airway management characteristics differed between the groups (S1 and S2 Tables). For example, compared to the patients with single intubation attempt, those with repeated attempts were more likely to be overweight or obese and intubated with a non-RSI method, and to have a predicted difficult intubation (≥1 modified LEMON score) (all P<0.05). Additionally, these patients were less likely to be intubated by emergency medicine resident or attending physician at the first attempt (P<0.001).

**Table 1:** Baseline characteristics of the study population, according to the outcome.

Variables	Overall (*n* = 3,097)	Post-intubation hypertension (*n* = 276)	No post-intubation hypertension (*n* = 2,821)	*P* value
Age (yr), median (IQR)	69 (53–78)	65 [51–76]	69 [54–79]	0.009
Age ≥65 years	1,805 (58.3)	142 (51.5)	1,663 (59.0)	0.02
Male sex	1,977 (64.0)	194 (70.3)	1,783 (63.2)	0.02
Body mass index category (kg/m^2^)				0.047
<18.5	366 (12.3)	22 (8.2)	344 (12.7)	
18.5–24.9	1,828 (61.3)	168 (62.7)	1,660 (61.2)	
25.0–29.9	614 (20.6)	55 (20.5)	559 (20.6)	
≥30	173 (5.8)	23 (8.6)	150 (5.5)	
Indication of intubation				
Medical encounters	2,608 (84.2)	221 (80.1)	2,387 (84.6)	0.04
Altered mental status	1,226 (39.6)	113 (40.9)	1,113 (39.5)	
Respiratory failure	920 (29.7)	59 (21.4)	861 (30.5)	
Shock	295 (9.5)	29 (10.5)	266 (9.4)	
Airway obstruction	129 (4.2)	17 (6.2)	112 (4.0)	
Other medical ^a^	38 (1.2)	3 (1.1)	35 (1.2)	
Trauma encounters	489 (15.8)	55 (19.9)	434 (15.4)	0.33
Head trauma	199 (6.4)	16 (5.8)	183 (6.5)	
Shock	96 (3.1)	11 (4.0)	85 (3.0)	
Other trauma ^b^	88 (2.8)	13 (4.7)	75 (2.7)	
Burn / inhalation	55 (1.8)	9 (3.3)	46 (1.6)	
Facial / neck trauma	51 (1.7)	6 (2.2)	45 (1.6)	

Abbreviation: IQR, interquartile range.

Data are expressed as number (percentage) unless otherwise indicated.

^a^ Defined as airway obstruction, asthma, anaphylaxis, and others.

^b^ Defined as multiple trauma and others.

**Table 2:** Airway management characteristics of the study population, according to the outcome.

Variables	Overall (*n* = 3,097)	Post-intubation hypertension (*n* = 276)	No post-intubation hypertension (*n* = 2,821)	*P* value
Method of indication				
RSI	1,675 (54.1)	189 (68.5)	1,486 (52.7)	<0.001
No RSI	1,422 (45.9)	87 (31.5)	1,335 (47.3)	
≥1 Modified LEMON	1,138 (50.6)	98 (46.5)	1,040 (51.0)	0.21
Device of intubation				0.01
Direct laryngoscope	1,998 (64.6)	157 (57.1)	1,841 (65.4)	
Video laryngoscope	1,062 (34.4)	113 (41.1)	949 (33.7)	
Other devices ^a^	32 (1.0)	5 (1.8)	27 (1.0)	
Premedication (fentanyl)	975 (31.5)	108 (39.1)	867 (30.7)	0.004
Premedication (fentanyl) dose (mcg/kg), median (IQR)	1.43 (1.00–1.82)	1.43 (1.00–1.67)	1.43 (1.00–1.82)	0.32
Sedative				0.002
Midazolam	1,198 (38.7)	133 (48.2)	1,065 (37.8)	
Propofol	578 (18.7)	40 (14.5)	538 (19.1)	
Ketamine	292 (9.4)	32 (11.6)	260 (9.2)	
Others ^b^	146 (4.7)	12 (4.4)	134 (4.8)	
None	879 (28.4)	59 (21.4)	820 (29.1)	
Sedative dose (mg/kg), median (IQR)				
Midazolam	0.07 (0.05–0.09)	0.07 (0.05–0.09)	0.07 (0.05–0.09)	0.95
Propofol	0.98 (0.71–1.31)	1.00 (0.67–1.67)	0.98 (0.71–1.27)	0.63
Ketamine	0.90 (0.71–1.04)	0.90 (0.73–1.36)	0.90 (0.71–1.03)	0.48
Neuromuscular blockades				<0.001
Rocuronium	1,601 (51.7)	176 (63.8)	1,425 (50.5)	
Succinylcholine	155 (5.0)	19 (6.9)	136 (4.8)	
Vecuronium	63 (2.0)	8 (2.9)	55 (2.0)	
None	1,278 (41.3)	73 (26.5)	1,205 (42.7)	
Specialty of the intubator				0.38
Transitional year resident ^c^	1,195 (38.8)	107 (38.8)	1,088 (38.8)	
Emergency medicine resident ^d^	1,049 (34.0)	102 (37.0)	947 (33.7)	
Emergency medicine attending physician ^e^	531 (17.2)	47 (17.0)	484 (17.2)	
Other specialty ^f^	308 (10.0)	20 (7.3)	288 (10.3)	
Number of intubation attempts	1 (1–2)	1 (1–2)	1 (1–2)	0.24
Repeated (≥2) intubation attempts	991 (32.0)	97 (35.1)	894 (31.7)	0.24

Abbreviations: RSI, rapid sequence intubation; IQR, interquartile range.

Data are expressed as number (percentage) unless otherwise indicated.

^a^ Defined as flexible bronchoscope and supraglottic devices.

^b^ Defined as administration of thiopental, diazepam, or combination with any of the included sedatives.

^c^ Defined as post-graduate year 1 or 2.

^d^ Defined as post-graduate years 3–5.

^e^ Defined as post-graduate years ≥6.

^f^ Defined as surgery, anesthesia, or pediatrics.

In the unadjusted analysis, there was no significant difference in the incidence between the patient groups (8.5% in the single intubation attempt vs. 9.8% in the repeated attempts; unadjusted P = 0.24: S2 Table). By contrast, after adjusting for potential confounders and patient clustering in the random-effects model, the patients who underwent repeated intubation attempts had a significantly higher risk of post-intubation hypertension (OR, 1.56; 95% CI, 1.11–2.18; adjusted P = 0.01; Table 3). Other factors that were associated with post-intubation hypertension were male sex, use of ketamine (compared to use of propofol), and use of neuromuscular blockers (all P<0.05). Comparison of premedication and sedatives according to the use of neuromuscular blockades was shown in S3 Table. The patients with neuromuscular blocker use were more likely to have received premedications (fentanyl) and sedatives (both P<0.05).

**Table 3:** Unadjusted and adjusted associations between the number of intubation attempts (primary exposure) and risk of post-intubation hypertension (outcome).

	Logistic regression model		Random-effect model	
Models and variables	OR (95%CI)	*P* value	OR (95%CI)	*P* value
**Unadjusted association**				
Number of attempts (≥2 vs 1)	1.17 (0.90–1.51)	0.24	1.31 (1.00–1.71)	0.052
**Adjusted association**				
Number of attempts (≥2 vs 1)	1.50 (1.08–2.09)	0.02	1.56 (1.11–2.18)	0.01
*Covariates*				
Age ≥65 years (vs.18-64 years)	0.72 (0.53–0.97)	0.03	0.71 (0.53–0.96)	0.06
Male sex	1.35 (0.97–1.86)	0.07	1.36 (0.99–1.89)	0.03
Body mass index category (kg/m^2^)				
<18.5	0.72 (0.42–1.21)	0.21	0.71 (0.42–1.20)	0.21
18.5–24.9	1 (reference)	-	1 (reference)	-
25.0–29.9	0.96 (0.66–1.39)	0.82	0.97 (0.67–1.41)	0.88
≥30	1.68 (0.96–2.92)	0.07	1.70 (0.97–2.97)	0.07
Indication				
Medical indications	1 (reference)	-	1 (reference)	-
Head and facial trauma	1.14 (0.64–2.04)	0.66	1.10 (0.61–1.98)	0.75
Other trauma	1.38 (0.84–2.25)	0.20	1.15 (0.68–1.92)	0.60
≥1 modified LEMON score (vs 0)	0.80 (0.59–1.08)	0.15	0.79 (0.58–1.08)	0.14
Device				
Direct laryngoscope	1 (reference)	-	1 (reference)	-
Video laryngoscope	0.98 (0.67–1.44)	0.92	0.85 (0.55–1.34)	0.49
Other devices ^a^	1.53 (0.33–7.03)	0.59	1.65 (0.36–7.59)	0.52
Premedication (fentanyl vs others)	1.07 (0.73–1.55)	0.73	1.04 (0.70–1.54)	0.86
Sedative				
Midazolam	1.51 (0.99–2.30)	0.054	1.38 (0.87–2.19)	0.17
Propofol	1 (reference)	-	1 (reference)	-
Ketamine	1.90 (1.10–3.28)	0.02	1.86 (1.04–3.32)	0.04
Others ^b^	0.82 (0.33–2.03)	0.66	0.73 (0.29–1.86)	0.51
None	1.28 (0.75–2.18)	0.37	1.16 (0.67–2.00)	0.60
Neuromuscular blockade use	2.25 (1.49–3.41)	<0.001	2.05 (1.33–3.17)	0.001
Specialty of intubator				
Transitional year resident ^c^	1 (reference)	-	1 (reference)	-
Emergency medicine resident ^d^	1.06 (0.73–1.54)	0.77	1.15 (0.77–1.70)	0.50
Emergency medicine attending physician ^e^	1.24 (0.79–1.94)	0.34	1.23 (0.78–1.94)	0.38
Other specialty ^f^	0.49 (0.25–0.98)	0.04	0.53 (0.26–1.05)	0.07

Abbreviations: OR, odds ratio; CI, confidence interval.

^a^ Defined as flexible bronchoscope and supraglottic devices.

^b^ Defined as administration of thiopental, diazepam, or combination with any of the included sedatives.

^c^ Defined as post-graduate year 1 or 2.

^d^ Defined as post-graduate years 3–5.

^e^ Defined as post-graduate years ≥6.

^f^ Defined as surgery, anesthesia, or pediatrics.

In the sensitivity analyses, the significant association between repeated intubation attempts and post-intubation hypertension persisted with the use of different definitions for repeated intubation attempts, and with limiting the samples to those with RSI and those with sedatives (Table 4). For example, compared to single intubation attempt, ≥3 intubation attempts were associated with a significantly higher risk of post-intubation hypertension with an adjusted OR of 1.93 (95%CI, 1.21–3.08; P = 0.006). In addition, even after adjusting for the dose of premedication and sedatives, the association between repeated intubation attempts and post-intubation hypertension remained significant (S4 Table).

**Table 4:** Sensitivity analysis using different cut-off for number of intubation attempts and different subgroups.

	Adjusted associations			
	Logistic regression		Random-effect model	
Models	OR (95%CI)	P-value	OR (95%CI)	P-value
**Different cut-off for number of intubation attempts** ^a^				
Number of attempts (≥3 vs ≤2)	1.69 (1.07–2.59)	0.03	1.74 (1.11–2.71)	0.02
Number of attempts (ordinal variable; OR per each incremental attempt)	1.20 (1.02–1.39)	0.02	1.26 (1.07–1.49)	0.006
Number of attempts (categorical variable)				
1	1 (reference)		1 (reference)	
2	1.34 (0.91–1.96)	0.14	1.39 (0.94–2.04)	0.10
≥3	1.85 (1.15–2.90)	0.01	1.93 (1.21–3.08)	0.006
**Subgroup analysis**				
Patients intubated with RSI ^b^				
Number of attempts ≥2 vs 1	1.62 (1.07–2.42)	0.02	1.67 (1.10–2.53)	0.02
Patients intubated with sedatives (excluded patients with no sedative use) ^a^				
Number of attempts ≥2 vs 1	1.72 (1.18–2.49)	0.005	1.80 (1.23–2.63)	0.003

Abbreviations: OR, odds ratio; CI, confidence interval; RSI, rapid sequence intubation.

^a^ Adjusted for age, sex, BMI, indication of intubation, modified LEMON score, device of intubation, premedication, sedatives, neuromuscular blockades, and specialty of the intubator.

^b^ Adjusted for age, sex, BMI, indication of intubation, modified LEMON score, device of intubation, premedication, sedatives, and specialty of the intubator.

## Discussion

In this analysis of the data from a 15-center prospective study of 3,097 patients who underwent emergency tracheal intubation in the ED, we found that approximately 9% of patients developed post-intubation hypertension. The previous ED literature has reported an intubation-related adverse event rate of approximately 12%-26%, not including post-intubation hypertension [1–3]. Thus, the 9% incidence post-intubation hypertension would add a substantial portion of adverse events related to airway management in the ED. The data also demonstrated that repeated intubation attempts are associated with a significantly higher risk of post-intubation hypertension. The significant association persisted across several different statistical assumptions. Other factors associated with the risk of post-intubation hypertension were male sex, use of ketamine (compared to the use of propofol), and use of neuromuscular blockades based on the random-effect model. To the best of our knowledge, this is the first effort that has examined the incidence of post-intubation hypertension and their risk factors in the ED.

The sparse literature has reported that post-intubation hypertension is associated with myocardial ischemia [12, 13], re-rupture of aneurysmal subarachnoid hemorrhage [14], and poor outcomes in patients with severe traumatic brain injury [15] in the non-ED setting, indicating that post-intubation hypertension–a consequence of an imbalance between sedation and stimulation (intubation)–should also be considered as an important adverse event. Despite the apparent clinical and research importance of post-intubation hypertension, only the non-ED literature has evaluated this outcome. For example, two retrospective studies in the prehospital setting reported that the incidence of post-intubation hypertension (defined as a >20% increase in sBP or mean arterial pressure from the baseline) was approximately 80% in both 97 patients with traumatic brain injury and 115 patients with trauma [33, 34]. Another retrospective single-center study of 57 patients with unplanned extubation who were emergently reintubated reported an incidence of post-intubation hypertension (defined as >20% increase in sBP from the baseline with sBP >160 mmHg) of 14% [23]. The between-study difference in the incidence of post-intubation hypertension are likely multifactorial, such as the difference in the patient populations. Indeed, while the population of prior prehospital study comprised of patients with traumatic brain injury–which is known to carry a high risk of hypertensive responses [29], our study had a smaller proportion of traumatic head injury (6%). Additionally, the differences in the study design (retrospective vs. prospective), setting (e.g., prehospital vs. ED), methods of BP measurements, and airway practices explain, at least in part, the apparent discrepancy of incidences.

Previous studies have shown the association between repeated intubation attempts and higher risk of intubation-related adverse events (not including post-intubation hypertension) across various clinical settings [5, 6, 8]. Consistently, the current study demonstrated, for the first time, the significant association between repeated attempts and elevated risk of post-intubation hypertension. There are several plausible underlying mechanisms of this novel association. Tracheal intubation attempt–a complex procedure consisting of direct laryngoscopy [35] and passage of the tracheal tube through the vocal cords and into the trachea [36]–induces sympathetic stimulation and increases in plasma catecholamines, thereby resulting in hemodynamic responses, such as tachycardia and elevation of blood pressure [37]. Particularly, studies have shown that an increasing force and duration at laryngoscopic attempts [38, 39] induce greater magnitude of these hemodynamic responses. The current study corroborates the earlier epidemiological and mechanistic studies, and extends them by demonstrating the associations between repeated intubation attempts and accelerated risks of post-intubation hypertension.

In the current study, other factors associated with the risk of post-intubation hypertension were male sex, use of ketamine (compared to use of propofol), and use of neuromuscular blockers. Patients with neuromuscular blockade use were more likely to have received sedatives, particularly benzodiazepines, which would have decreased the incidence of post-intubation hypertension. Regardless, because the observed association between the neuromuscular blockade use and outcome was independent from other covariates (including sedative use), it cannot be explained by the difference in sedative use. Alternatively, it is possible that the use (and choice) of neuromuscular blockades might have been related to other factors (e.g., individual intubator’s skills, competencies, and practice patterns) that are also associated with the occurrence of post-intubation hypertension.

This study has several potential limitations. First, passive surveillance introduces a self-report bias, and possible underestimation of the incidence of post-intubation hypertension. It is challenging to record real-time data accurately in the ED when emergency intubation is required. However, we used a previously applied standardized data collection system with a capture rate (97%) [9, 40]. Second, the observed associations might have been biased by unmeasured confounders, such as patient’s baseline cardiopulmonary reserve and between-hospital differences in the airway practice. Yet, the significant associations between repeated intubation attempts and post-intubation hypertension persisted after accounting for patient clustering within hospitals. Third, the current study did not measure post-ED clinical outcomes (e.g., inhospital mortality, neurological outcomes). Therefore, we were unable to assess the potential heterogeneity in the impact of the observed associations between different disease conditions. For example, in patients with traumatic brain injury, post-intubation hypertension is–through improved brain perfusion–potentially favorable while this intubation-related adverse event may be harmful in other patient groups (e.g., patients with aortic dissection). Fourth, our observational data do not have the information on the methods used for blood pressure measurements–i.e., non-invasive vs. invasive monitoring. However, non-invasive monitoring is well correlated with the invasive blood pressure [41], and widely used in clinical settings. Fifth, while the multivariable models rigorously adjusted for the type of medications used, it is possible that the causal inference is confounded by other clinical factors that affected clinical decision making. Finally, the study cohort comprised patients in academic EDs across Japan; our inferences therefore might not be generalizable to other ED settings. However, the association is plausible and has been demonstrated in the other clinical settings (e.g., operating rooms), supporting the validity of our inferences.

## Conclusions

In conclusion, in this analysis of a 15-center prospective study of 3,097 patients undergoing intubation in the ED, approximately 9% developed post-intubation hypertension. We also found that repeated intubation attempts were associated with a significantly higher risk of post-intubation hypertension. The observed association persisted across several different analytic assumptions. The multivariable models also demonstrated that male sex, the use of ketamine, and neuromuscular blockades were associated with a higher risk of post-intubation hypertension. For clinicians, as post-intubation hypertension is known to be related to worse clinical outcomes, our observations not only underscore the importance of recognition and monitoring for this intubation-related adverse event, but also lend additional significant support to the concept of first-pass success in the ED.

## Supporting information

S1 Table Baseline characteristics according to the primary outcome. (DOCX)

S2 Table Airway management characteristics according to the primary outcome. (DOCX)

S3 Table Comparison of premedication and sedatives use, according to neuromuscular blockade use. (DOCX)

S4 Table Adjusted associations between the number of intubation attempts and risk of post-intubation hypertension, adjusting for premedication and sedatives dosage in addition to other covariates. (DOCX)
