# Supplementary material for: The incidence of post-intubation hypertension and association with repeated intubation attempts in the emergency department
Source: PLoS One. 2019 Feb 11;14(2):e0212170. doi: 10.1371/journal.pone.0212170 (PMC6370241; doi:10.1371/journal.pone.0212170)
Supplement: S1 Table — (DOCX) [file pone.0212170.s001.docx]

**S1 Table. Baseline characteristics of the patients according to the frequency of intubation attempts**

| **Variables** | **Overall**  (*n* = 3,097) | **Single attempt**  (*n* = 2,106) | **Repeated (≥2) attempts**  (*n* = 991) | ***P* value** |
| --- | --- | --- | --- | --- |
| Age (yr), median (IQR) | 69 (53-78) | 69 (55-79) | 68 (52-78) | 0.03 |
| Age ≥65 years | 1,805 (58.3) | 1,251 (59.4) | 554 (55.9) | 0.07 |
| Male sex | 1,977 (64.0) | 1,324 (62.9) | 653 (65.9) | 0.10 |
| Body mass index category (kg/m^2^) | |  |  | 0.003 |
| <18.5 | 366 (12.3) | 266 (13.2) | 100 (10.4) |  |
| 18.5-24.9 | 1,828 (61.3) | 1,254 (62.1) | 574 (59.6) |  |
| 25.0-29.9 | 614 (20.6) | 396 (19.6) | 218 (22.6) |  |
| ≥30 | 173 (5.8) | 102 (5.1) | 71 (7.4) |  |
| Indication of intubation |  |  |  |  |
| Medical encounters | 2,608 (84.2) | 1,782 (84.6) | 826 (83.4) | <0.001 |
| Altered mental status | 1,226 (39.6) | 811 (38.5) | 415 (41.9) |  |
| Respiratory failure | 920 (29.7) | 637 (30.3) | 283 (28.6) |  |
| Shock | 295 (9.5) | 230 (10.9) | 65 (6.6) |  |
| Airway obstruction | 129 (4.2) | 73 (3.5) | 56 (5.7) |  |
| Other medical ^a^ | 38 (1.2) | 31 (1.5) | 7 (0.7) |  |
| Trauma encounters | 489 (15.8) | 324 (15.4) | 165 (16.7) | 0.003 |
| Head trauma | 199 (6.4) | 116 (5.5) | 83 (8.4) |  |
| Shock | 96 (3.1) | 79 (3.8) | 17 (1.7) |  |
| Other trauma ^b^ | 88 (2.8) | 59 (2.8) | 29 (2.9) |  |
| Burn / inhalation | 55 (1.8) | 41 (2.0) | 14 (1.4) |  |
| Facial / neck trauma | 51 (1.7) | 29 (1.4) | 22 (2.2) |  |

Abbreviation: IQR, interquartile range.

Data are expressed as number (percentage) unless otherwise indicated.

^a^ Defined as airway obstruction, asthma, anaphylaxis, and others.

^b^ Defined as multiple trauma and others.
