# Supplementary material for: The incidence of post-intubation hypertension and association with repeated intubation attempts in the emergency department
Source: PLoS One. 2019 Feb 11;14(2):e0212170. doi: 10.1371/journal.pone.0212170 (PMC6370241; doi:10.1371/journal.pone.0212170)
Supplement: S2 Table — (DOCX) [file pone.0212170.s002.docx]

**S2 Table. Airway management characteristics according to the frequency of intubation attempts**

| **Variables** | **Overall**  (*n* = 3,097) | **Single attempt**  (*n* = 2,106) | **Repeated (≥2) attempts**  (*n* = 991) | ***P* value** |
| --- | --- | --- | --- | --- |
| Method of intubation |  |  |  | <0.001 |
| RSI | 1,675 (54.1) | 1,261 (59.9) | 414 (41.8) |  |
| No RSI | 1,422 (45.9) | 845 (40.1) | 577 (58.2) |  |
| ≥1 modified LEMON score | 1,138 (50.6) | 684 (44.7) | 454 (63.1) | <0.001 |
| Device of intubation |  |  |  | <0.001 |
| Direct laryngoscope | 1,998 (64.6) | 1,278 (60.1) | 720 (72.7) |  |
| Video laryngoscope | 1,062 (34.4) | 803 (38.2) | 259 (26.2) |  |
| Other devices ^a^ | 32 (1.0) | 21 (1.0) | 11 (1.1) |  |
| Premedication (fentanyl) | 975 (31.5) | 736 (35.0) | 239 (24.1) | <0.001 |
| Premedication (fentanyl) dose (mcg/kg), median (IQR) | 1.43 (1.00-1.82) | 1.45 (1.00-1.82) | 1.43 (0.98-1.67) | 0.14 |
| Sedative |  |  |  | <0.001 |
| Midazolam | 1,198 (38.7) | 856 (40.7) | 342 (34.5) |  |
| Propofol | 578 (18.7) | 390 (18.6) | 188 (19.0) |  |
| Ketamine | 292 (9.4) | 219 (10.4) | 73 (7.4) |  |
| Others ^b^ | 146 (4.7) | 106 (5.0) | 40 (4.0) |  |
| None | 879 (28.4) | 531 (25.3) | 348 (35.1) |  |
| Sedative dose (mg/kg), median (IQR) | |  |  |  |
| Midazolam | 0.07 (0.05-0.09) | 0.06 (0.05-0.09) | 0.07 (0.05-0.10) | 0.02 |
| Propofol | 0.98 (0.71-1.31) | 1.00 (0.71-1.33) | 0.89 (0.68-1.20) | 0.17 |
| Ketamine | 0.90 (0.71-1.04) | 0.89 (0.71-1.04) | 0.91 (0.71-1.07) | 0.96 |
| Neuromuscular blockade |  |  |  | <0.001 |
| Rocuronium | 1,601 (51.7) | 1,208 (57.4) | 393 (39.7) |  |
| Succinylcholine | 155 (5.0) | 108 (5.1) | 47 (4.7) |  |
| Vecuronium | 63 (2.0) | 41 (2.0) | 22 (2.2) |  |
| None | 1,278 (41.3) | 749 (35.6) | 529 (53.4) |  |
| Specialty of intubator |  |  |  | <0.001 |
| Transitional year resident ^c^ | 1,195 (38.8) | 683 (32.6) | 512 (51.9) |  |
| Emergency medicine resident ^d^ | 1,049 (34.0) | 802 (38.3) | 247 (25.0) |  |
| Emergency medicine  attending physician ^e^ | 531 (17.2) | 416 (19.9) | 115 (11.7) |  |
| Other specialty ^f^ | 308 (10.0) | 195 (9.3) | 113 (11.5) |  |
| Post-intubation hypertension | 276 (8.9) | 179 (8.5) | 97 (9.8) | 0.24 |

Abbreviations: RSI, rapid sequence intubation; IQR, interquartile range.

Data are expressed as number (percentage) unless otherwise indicated.

^a^ Defined as flexible bronchoscope and supraglottic devices.

^b^ Defined as administration of thiopental, diazepam, or combination with any of the included sedatives.

^c^ Defined as post-graduate year 1 or 2.

^d^ Defined as post-graduate years 3-5.

^e^ Defined as post-graduate years ≥6.

^f^ Defined as surgery, anesthesia, or pediatrics.
