# Supplementary material for: The incidence of post-intubation hypertension and association with repeated intubation attempts in the emergency department
Source: PLoS One. 2019 Feb 11;14(2):e0212170. doi: 10.1371/journal.pone.0212170 (PMC6370241; doi:10.1371/journal.pone.0212170)
Supplement: S3 Table — (DOCX) [file pone.0212170.s003.docx]

**S3 Table.** **Comparison of premedication and sedative use, according to neuromuscular blockade use**

| **Variables** | **NMB use**  (n=1,816) | **No NMB use**  (n=1,281) | ***P* value** |
| --- | --- | --- | --- |
| Premedication (fentanyl) | 728 (40.1) | 247 (19.3) | <0.001 |
| Premedication (fentanyl) dose  (mcg/kg), median (IQR) | 1.47 (1.09-1.82) | 1.33 (0.90-1.75) | 0.004 |
| Sedative |  |  |  |
| Midazolam | 948 (52.2) | 250 (19.5) | <0.001 |
| Propofol | 379 (20.9) | 199 (15.6) | <0.001 |
| Ketamine | 241 (13.3) | 51 (4.0) | <0.001 |
| Others | 98 (5.4) | 48 (3.8) | 0.03 |
| None | 150 (8.3) | 729 (57.1) | <0.01 |
| Sedative dose (mg/kg), median  (IQR) |  |  |  |
| Midazolam (mg/kg) | 0.065 (0.05-0.09) | 0.071 (0.05-0.10) | 0.006 |
| Propofol (mg/kg) | 0.95 (0.71-1.30) | 1.00 (0.71-1.28) | 0.52 |
| Ketamine (mg/kg) | 0.89 (0.71-1.00) | 0.97 (0.70-1.20) | 0.28 |

Abbreviations: NMB, Neuromuscular blockade; IQR, interquartile range.

Data are expressed as number (percentage) unless otherwise indicated.
