# Supplementary material for: The incidence of post-intubation hypertension and association with repeated intubation attempts in the emergency department
Source: PLoS One. 2019 Feb 11;14(2):e0212170. doi: 10.1371/journal.pone.0212170 (PMC6370241; doi:10.1371/journal.pone.0212170)
Supplement: S4 Table — (DOCX) [file pone.0212170.s004.docx]

**S4 Table. Adjusted associations between the number of intubation attempts and risk of post-intubation hypertension, adjusting for premedication and sedatives dosage in addition to other covariates**

|  | **Logistic regression model** | | **Random-effect model** | |
| --- | --- | --- | --- | --- |
| **Models and variables** | OR (95%CI) | *P* value | OR (95%CI) | *P* value |
| **Adjusted association** |  |  |  |  |
| Number of attempts (≥2 vs 1) | 1.50 (1.07-2.08) | 0.02 | 1.56 (1.11-2.18) | 0.01 |
| *Covariates* |  |  |  |  |
| Age ≥65 years (vs.18-64 years) | 0.74 (0.55-1.00) | 0.051 | 0.74 (0.55-1.00) | 0.047 |
| Male sex | 1.36 (0.99-1.89) | 0.06 | 1.37 (0.99-1.90) | 0.06 |
| Body mass index category (kg/m^2^) | |  |  |  |
| <18.5 | 0.70 (0.40-1.16) | 0.17 | 0.70 (0.41-1.18) | 0.18 |
| 18.5-24.9 | 1 (reference) | - | 1 (reference) | - |
| 25.0-29.9 | 0.99 (0.67-1.42) | 0.94 | 0.99 (0.68-1.45) | 0.97 |
| ≥30 | 1.72 (0.96-2.95) | 0.07 | 1.72 (0.98-3.02) | 0.06 |
| Indication |  |  |  |  |
| Medical indications | 1 (reference) | - | 1 (reference) | - |
| Head and facial trauma | 1.14 (0.62-1.99) | 0.65 | 1.02 (0.50-2.10) | 0.95 |
| Other trauma | 1.38 (0.83-2.22) | 0.20 | 0.91 (0.51-1.64) | 0.76 |
| ≥1 modified LEMON score (vs 0) | 0.79 (0.58-1.08) | 0.14 | 0.79 (0.58-1.08) | 0.15 |
| Device |  |  |  |  |
| Direct laryngoscope | 1 (reference) | - | 1 (reference) | - |
| Video laryngoscope | 1.00 (0.67-1.44) | 0.99 | 0.84 (0.53-1.32) | 0.45 |
| Other devices ^a^ | 1.59 (0.24-5.94) | 0.57 | 1.67 (0.36-7.70) | 0.51 |
| Premedication (fentanyl) dose (mcg/kg) | 1.02 (0.83-1.24) | 0.82 | 1.08 (0.72-1.60) | 0.70 |
| Sedative dose (mg/kg) |  |  |  |  |
| Midazolam | 3.98 (0.40-27.12) | 0.22 | 3.82 (0.48-30.63) | 0.21 |
| Propofol | 1.04 (0.75-1.40) | 0.80 | 1.14 (0.83-1.59) | 0.42 |
| Ketamine | 1.72 (1.12-2.58) | 0.01 | 1.77 (1.16-2.70) | 0.008 |
| Neuromuscular blockade use | 2.25 (1.49-3.41) | <0.001 | 1.99 (1.34-2.95) | 0.001 |
| Specialty of intubator |  |  |  |  |
| Transitional year resident ^b^ | 1 (reference) | - | 1 (reference) | - |
| Emergency medicine resident ^c^ | 1.06 (0.73-1.54) | 0.75 | 1.16 (0.78-1.72) | 0.45 |
| Emergency medicine attending physician ^d^ | 1.22 (0.77-1.89) | 0.39 | 1.21 (0.76-1.91) | 0.43 |
| Other specialty ^e^ | 0.49 (0.23-0.93) | 0.03 | 0.52 (0.26-1.04) | 0.07 |

Abbreviations: OR, odds ratio; CI, confidence interval.

^a^ Defined as flexible bronchoscope and supraglottic devices.

^b^ Defined as post-graduate year 1 or 2.

^c^ Defined as post-graduate years 3-5.

^d^ Defined as post-graduate years ≥6.

^e^ Defined as surgery, anesthesia, or pediatrics.
